# Revisiting Mehrotra and Nichani’s Corner Detection Method for Improvement with Truncated Anisotropic Gaussian Filtering

**DOI:** 10.3390/s23208653

**Published:** 2023-10-23

**Authors:** Baptiste Magnier, Khizar Hayat

**Affiliations:** 1 Euromov Digital Health in Motion, Univ Montpellier, IMT Mines Ales, Ales, France; 2 College of Arts and Sciences, University of Nizwa, Nizwa 616, Oman; khizar.hayat@unizwa.edu.om

**Keywords:** corner detection, truncated Gaussian, half edges, oriented Gaussian, anisotropic Gaussian, first derivative of the Gaussian

## Abstract

In the early 1990s, Mehrotra and Nichani developed a filtering-based corner detection method, which, though conceptually intriguing, suffered from limited reliability, leading to minimal references in the literature. Despite its underappreciation, the core concept of this method, rooted in the half-edge concept and directional truncated first derivative of Gaussian, holds significant promise. This article presents a comprehensive assessment of the enhanced corner detection algorithm, combining both qualitative and quantitative evaluations. We thoroughly explore the strengths, limitations, and overall effectiveness of our approach by incorporating visual examples and conducting evaluations. Through experiments conducted on both synthetic and real images, we demonstrate the efficiency and reliability of the proposed algorithm. Collectively, our experimental assessments substantiate that our modifications have transformed the method into one that outperforms established benchmark techniques. Due to its ease of implementation, our improved corner detection process has the potential to become a valuable reference for the computer vision community when dealing with corner detection algorithms. This article thus highlights the quantitative achievements of our refined corner detection algorithm, building upon the groundwork laid by Mehrotra and Nichani, and offers valuable insights for the computer vision community seeking robust corner detection solutions.

## 1. Introduction and Motivations

Corner detection plays a pivotal role in the realm of computer vision, serving as a foundational cornerstone for a multitude of image-processing tasks, including vision-based recognition. Corner points embody stable features with well-defined characteristics, making them robust points of interest [1,2,3,4,5,6,7,8,9]. Their accurate detection is quintessential to computer vision sensors in the identification and localization of key points with unique visual features, for example, for target detection [10]. Unquestionably, corners are distinctive image locations where intensity variations occur in multiple directions, making them robust and invariant to transformations like rotation and scale changes. This may prove particularly useful in numerous applications such as image-guided surgery [11]. Additionally, they are critical for identifying salient features and object boundaries in images, making their ascertainment inevitable for such tasks as image registration, object tracking, and image stitching. Beyond these immediate applications, the extraction and characterization of corners have paved the way for remarkable advancements in feature matching, leading to breakthroughs in object recognition and 3D reconstruction. The application of corner detection extends to various domains, including autonomous navigation, robotics, augmented reality, and facial recognition systems. In recent years, corner detection algorithms have gained widespread recognition and practical relevance in engineering domains. These algorithms serve as fundamental building blocks for a variety of applications, including robotics for simultaneous localization and mapping (SLAM [12,13]), advanced driver assistance systems—ADAS (https://www.synopsys.com/automotive/what-is-adas.html, accessed on 17 October 2023)—in automotive engineering, structural analysis in civil engineering, especially dealing with cracks [14], and feature extraction in medical imaging. The versatility and real-world utility of corner detection algorithms make them indispensable tools in modern engineering, and this paper explores their significance and applications, providing readers with a comprehensive view of their engineering impact.

In the last part of the 20th century, corner [1,15] and edge detection [16,17,18] witnessed several pioneering and milestone works that laid the foundation for modern computer vision. In this context, an under-appreciated yet influential work is by Mehrotra and Nichani [19], which provides valuable insights into accurately identifying corners in images. They relied on the concept of half-edges to propose two algorithms on the basis of first- and second-directional derivatives.

It is crucial in corner detection algorithms to identify the salient corner points with high precision and robustness. By quantifying the level of corner-like structures at each pixel, ‘*cornerness*’ distinguishes corners from other image features, such as edges and flat regions [20,21]. The appropriate choice of such measures directly impacts the algorithm’s performance, influencing its sensitivity to noise and ability to handle scale and orientation variations. There are different approaches to determining the cornerness measure by direct computation using filtering techniques; a recent review [22] details these measures, and can be further complemented with [23,24].

Gaussian kernels are commonly employed due to their efficacy in edge detection. However, their limitations become apparent when dealing with blurred or noisy images, as well as when detecting edges around corners and small objects. These limitations are particularly noticeable when using isotropic kernels, such as those employed in the traditional Canny edge detector [16]. In order to enhance detection precision, elongated oriented filters were devised to strike a more favorable balance between noise reduction and localization accuracy [25,26,27,28]. Elsewhere, half-filters enable the estimation of contour information across multiple directions, spanning to completion [19,29,30], unlike fully oriented Gaussians that are symmetrical relying on information up to $180^{\circ }$ [26,27,28].

In this work, while relying on the truncated first Gaussian derivative method in [19], we propose introducing anisotropy to the underlying filters. Oriented isotropic filters were used in the original method that has the disadvantage of not being so reliable against acute-angled corners for being symmetric. We aim to improve the original method with the truncated first anisotropic Gaussian derivative. For the sake of comparison, three classical corner detection algorithms have been chosen, namely: Kitchen and Rosenfeld [31], Harris and Stephen [32], Shi and Tomasi [33]. They are outlined in Section 4 and these compared methods are also detailed in the Tab. I in [22] and in the Tab. II in [23] with the filtering formulas, parameters, and description. The reason for choosing these classical methods is that the reference article also belongs almost to the same period. In this context, the use of classical corner detection methods as benchmarks is a valid and informative approach, especially when working on improving older techniques. It provides a historical perspective and helps readers understand the significance of our contributions. While newer corner detection methods [34,35,36] based on deep learning and other advanced techniques have emerged (the introduction by Zhang et al. [24] is a nice compendium), the classical algorithms by image filtering like Harris [32] and Shi-Tomasi [33] remain relevant because they provide a solid baseline for performance and continue to be effective in many practical applications. Additionally, they are often the first choice when simplicity, efficiency, and well-understood behavior are critical.

The rest of the paper is organized as follows. The foundation method is introduced in Section 2 followed by a theoretical analysis in Section 3 related to the proposed improvements. Section 4 details the experimental evaluation and results. Finally, Section 5 concludes this paper.

## 2. The Original Corner Detection Method of Mehrotra and Nichani

Originally, Mehrotra and Nichani in [19] proposed two corner detection algorithms: one was based on the first directional derivative of Gaussian and the other concerned the second directional derivative of Gaussian. For this study, we focus on the first derivative of the Gaussian function for detection, which is simpler to establish (avoiding particularly zero crossing calculations). Indeed, in the original paper, corners are defined as the junction point of (at least) two straight-line edges, oriented in two different, but not opposite, directions. Consequently, the main idea of this algorithm is to detect half edges by the means of truncated first directional derivative of Gaussian. The purpose of this detector is to compute both the corner angle and the edge directions tied to this corner.

### 2.1. Truncated Gaussian Filters

Gaussian half-filters are user-friendly and dependable for image analysis. These oriented filters, directed in various desired directions around each pixel, prove valuable for contour detection and precise orientation extraction, even in the presence of high image noise. The oriented half-filters, as presented in references [19,29], share similarities with, and perhaps even draw inspiration from, the well-known and widely used ’steerable filters’ introduced by Freeman and Adelson [37]. These filters employ a full 2D Gaussian with isotropic characteristics, where the calculation of the gradient’s magnitude corresponds to the energy along the direction of the maximum response of the filter. Freeman and Adelson’s work demonstrates that the first derivative of the 2D directional Gaussian $G_{\sigma ,\theta }$, steered at an angle θ, can be synthesized through a linear combination of the derivatives of the fundamental isotropic Gaussian with respect to the *x* and *y* axes:

(1) $$G_{\sigma ,\theta }\left(x,y\right)=\cos\left(\theta \right)\cdot \frac{\partial G_{\sigma }}{\partial x}\left(x,y\right)+\sin\left(\theta \right)\cdot \frac{\partial G_{\sigma }}{\partial y}\left(x,y\right),$$

where the pixel’s coordinates are denoted as (x,y), and σ represents the standard deviation of the Gaussian $G_{\sigma }$.

In the context of digital images, it is common for a single pixel to be traversed by multiple contours. For instance, consider a pixel situated at a corner where multiple directions intersect. These directions can be effectively estimated using half-filters. These filters prove valuable and efficient in applications such as image restoration through partial differential equations (PDE) [30], corner detection [38], or descriptors [39], often in combination with other image processing techniques. Mehrotra and Nichani proposed to utilize directly the response of these truncated filters for corner detection [19]. Considering a 2D isotropic Gaussian filter *G* of standard deviation σ:

(2) $$G_{\sigma }\left(x,y\right)=\frac{1}{2\pi \sigma^{2}}\cdot e^{-\frac{x^{2}+y^{2}}{2\sigma^{2}}}\;\;,\;\mathrm{with}\;\sigma \;\in \;R_{+}^{*}\;\mathrm{and}\;\left(x,y\right)\in R^{2}\;\;,$$

its first derivative is calculated by:

(3) $$G_{\sigma }^{\prime }\left(x,y\right)=\frac{\partial G_{\sigma }}{\partial x}\left(x,y\right)=-\frac{x}{\sigma^{2}}\cdot G_{\sigma }\left(x,y\right)=\frac{-x}{2\pi \sigma^{4}}\cdot e^{-\frac{x^{2}+y^{2}}{2\sigma^{2}}}.$$

Consequently, the truncated Gaussian derivative $HG^{\prime }$ proposed by Mehrotra and Nichani can be written as:

(4) $$HG_{\sigma }^{\prime }\left(x,y\right)=H\left(y\right)\cdot \left(-\frac{x}{2\pi \sigma^{4}}\cdot e^{-\frac{x^{2}+y^{2}}{2\sigma^{2}}}\right),$$

where H represents the Heaviside step function:

(5) $$H\left(s\right)=\left\{\begin{array}{cc} 1, & \;\mathrm{if}\;\;s>0,\\ 0, & \;\mathrm{elsewhere}. \end{array}\right.$$

For illustration purposes, some $HG_{\sigma }^{\prime }$ filters are displayed in Table 1, as a function of the parameter σ. Note that each of these filters represents a set of rotated versions with various angles (0≤θ<360), see details in [29].

### 2.2. The Corner Detection Process

Mehrotra and Nichani [19] view a corner as “*the intersection of two half-edges, oriented in 2 different directions, which are not 180° apart*”. Hence, their strategy hinges upon the detection of “half-edges” while relying on a single orientation instead of the opposing directions. To this end, they propose two algorithms for not only locating the corners but also their angles/orientations; one on the basis of the first and the other based on the second directional Derivative of Gaussian.

As already stated, our focus is the first derivative version for its simplicity. The algorithm creates, for each possible half-edge orientation, a set of convolution masks $HG_{\sigma }^{\prime }$. The number of these masks is also pre-decided based on the subdivision of the orientation interval [O,360−Δθ] (in degrees), such that $\frac{360^{\circ }}{\Delta \theta }$ corresponds to the number of directions covered by the filter $HG_{\sigma }^{\prime }$. After treating the image with each mask separately, the algorithm extracts for each pixel the edge orientations ($\theta_{1}$, $\theta_{2}$) pairs and the gradient magnitude |∇I|, corresponding to the two most responsive masks, based on a pre-decided but preferably high threshold:

(6) $$\left\{\begin{array}{ccc} \left|\nabla I|(x,y\right) & = & \max_{\theta \in [0,360^{\circ }[}\;\;\;\;I*HG_{\sigma }^{\prime }\left(x,y\right)-\min_{\theta \in [0,360^{\circ }[}I*HG_{\sigma }^{\prime }\left(x,y\right),\\ \theta_{1}\left(x,y\right) & = & \underset{\theta \in [0,360^{\circ }[}{\mathrm{argmax}}\left(I*HG_{\sigma }^{\prime }\left(x,y\right)\right),\\ \theta_{2}\left(x,y\right) & = & \underset{\theta \in [0,360^{\circ }[}{\mathrm{argmin}}\left(I*HG_{\sigma }^{\prime }\left(x,y\right)\right),\\ \eta (x,y) & = & \frac{\theta_{1}\left(x,y\right)+\theta_{2}\left(x,y\right)}{2}, \end{array}\right.$$

where ‘∗’ represents the convolution product.

Here, η represents the bisector between the 2 directions ($\theta_{1}$, $\theta_{2}$), which is perpendicular to the edge orientation in the image. Consequently, edges can be extracted by non-maxima suppression (NMS) in the η direction (detailed in [18]). Theoretically, a corner point is also an edge point in the image. In Mehrotra and Nichani’s method, non-edge points are first removed, as detailed in the following.

**Figure 1:**
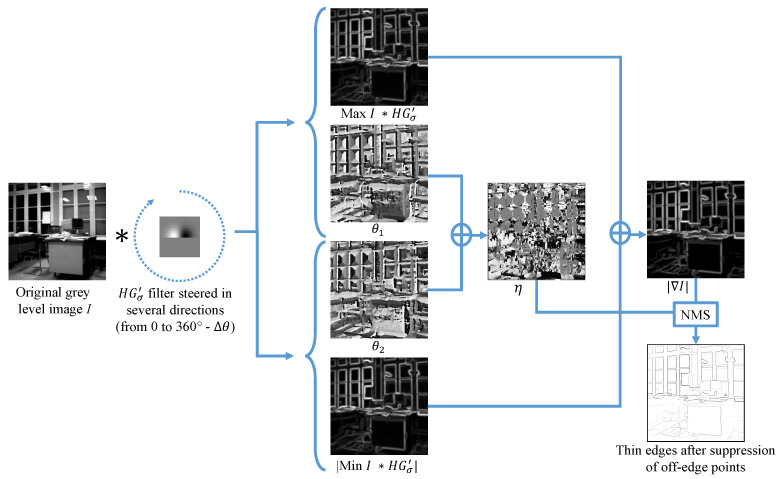
Off-edge points removal: non-maxima pixels of |∇I| are removed in the direction of η, obtaining thin edges (the thin edge image is inverted for better visualization). Corner points are selected among these thin edges as a function of $\theta_{1}$ and $\theta_{2}$ directions. Here, the dotted arrow represents the filter rotation whereas the solid ones are tied to the operation results in the Equation (6).

The responsive pixels can be partitioned into four categories among which one corresponds to corner points the algorithm wants to exclusively detect. The other three need to be eliminated as follows:

1. Off-edge points: A point is identified, and subsequently eliminated, as an off-edge point if its response is significantly subdued in comparison to its neighborhood, by using NMS in the η direction (calculated in Equation (6)). Figure 1 illustrates this process.
2. Non-corner edge points: Two adjacent responsive points are easily non-corner edge points if their orientations differ by π. These were the criteria adopted for their elimination in the algorithm:
  (7) $$\beta \left(x,y\right)=|\theta_{1}\left(x,y\right)-\theta_{2}\left(x,y\right)|.$$
  If $\beta \left(x,y\right)>180^{\circ }$, $\beta \left(x,y\right)=360^{\circ }-\beta \left(x,y\right)$, obtaining precisely the corner angle allows a selection of specific corners.
3. Off-corner points: These are eliminated by retaining only those points whose response is maximum in their neighborhood (NMS in a rectangular or circular mask).

**Figure 2:**
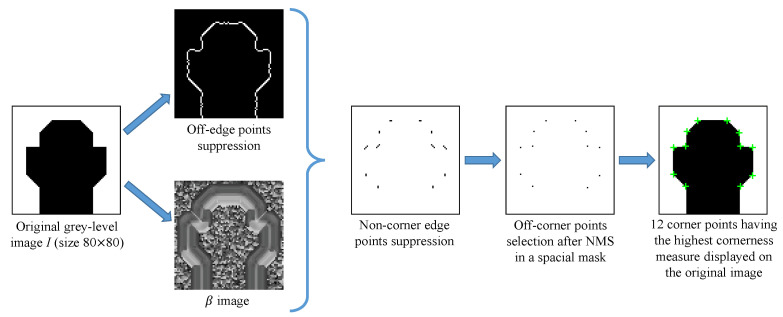
The overall corner detection process: *(i)* off-edge points are removed while the angle β is computed (corresponding to the edge direction crossing each pixel, see Equation (7)), then *(ii)* non-corner points are suppressed before *(iii)* applying a spacial non-maxima suppression (NMS) to extract corner points (corner points appear with green ‘+’ on the left). Note that the images in the middle are inverted (negatives) for a better visualization.

After eliminating off-edge, non-corner edge, and off-corner points, what is left out are the corner points. Figure 2 illustrates the elimination of off-edge points, non-corner edge points, and off-corner points via a detailed flow diagram.

**Figure 3:**
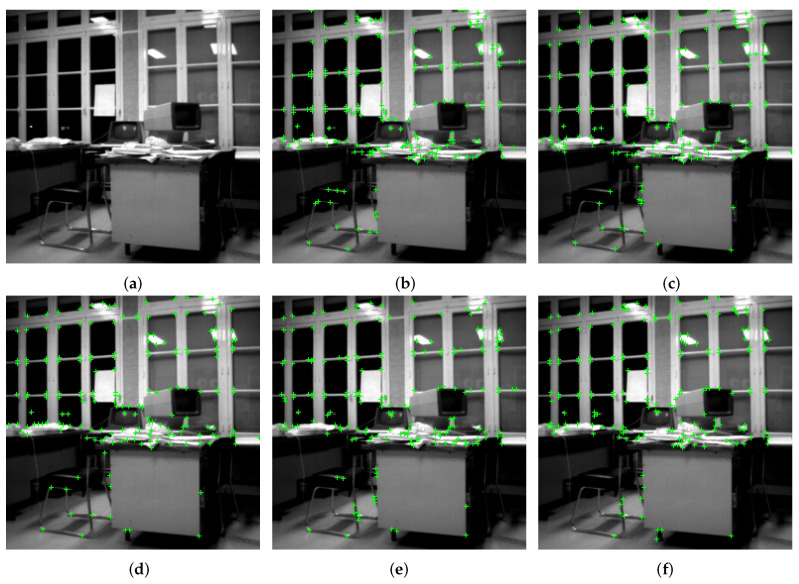
Comparison of corner detection on a real image. Here, 180 corners (represented by green ‘+’) are extracted having the highest cornerness measure, and the area for NMS is a square of 5 × 5. (**a**) Original image, 256 × 256 pixel size. (**b**) Kitchen and Rosenfeld [31]. (**c**) Shi and Tomasi [33], σ=1 for the tensor. (**d**) Harris and Stephen [32], σ=1 for the tensor. (**e**) Mehrotra and Nichani [19], σ=1 for $HG_{\sigma }^{\prime }$. (**f**) Proposed method, σ=1 and μ=3 for $HGK_{\sigma ,\mu }$.

Mehrotra and Nichani [19] utilized an image similar to those in displayed in Figure 2 such that the selection angles complying $90^{\circ }\leq \beta \leq 150^{\circ }$, make corners extraction fairly easier using the oriented truncated first derivative of isotropic Gaussian. Nevertheless, the original method was not deeply tested and compared. On the one hand, the method was only tested regarding synthetic images, which do not contain sharp corners, i.e., $\beta \leq 90^{\circ }$. On the other hand, the basic idea of this technique (with the two half edges) is obvious and seems promising, but it does not seem efficient in relating to real images, containing both blur and noise (caused by the sensor or movements). To illustrate, the corners detected in Figure 3e are not always satisfactorily well localized (examples are some of the detected corners along the window bars). Usually, compared to the classical corner detection methods of Shi and Tomasi [33] and Harris and Stephen [32] (presented in Figure 3c,d, respectively), the Mehrotra and Nichani’s technique remains less efficient. For those reasons, we propose customizing the filter, in order to cope with image degradation and better detect acute corners, in a way to narrow down the filter to maintain the most robust precision possible, as detailed in the following section.

## 3. Improving the Method with Anisotropic Gaussian Kernels

Oriented filters were devised with the purpose of capturing variations in gray intensity from multiple directions [26,37]. In this context, elongated Gaussians have proven to be effective in accurately detecting large linear structures [27,28,40]. The concept was especially extended in [28], where a given kernel was decomposed optimally into a set comprising the basis filters, approximating an Anisotropic Gaussian Kernel (AGK). The AGK filter is constructed by convolving two 1D Gaussian filters using the convolution operation denoted as “∗”:

(8) $$AGK_{\sigma ,\mu }\left(x,y\right)=\frac{1}{\sqrt{2\pi }\sigma }\cdot e^{-\frac{x^{2}+y^{2}}{2\sigma^{2}}}*\frac{1}{\sqrt{2\pi }\mu }\cdot e^{-\frac{x^{2}+y^{2}}{2\mu^{2}}}=\frac{1}{2\pi \cdot \sigma \cdot \mu }\cdot e^{-\frac{1}{2}\left(\frac{x^{2}}{\sigma^{2}}+\frac{y^{2}}{\mu^{2}}\right)}.$$

The parameter σ represents the Gaussian scale, whereas μ pertains to anisotropy. Accordingly, the First Order Anisotropic Gaussian Kernel can be constructed based on the AGK as follows:

(9) $$FOAGK_{\sigma ,\mu }\left(x,y\right)=\frac{\partial }{\partial x}\frac{1}{2\pi \cdot \sigma \cdot \mu }\cdot e^{-\frac{1}{2}\left(\frac{x^{2}}{\sigma^{2}}+\frac{y^{2}}{\mu^{2}}\right)}=\frac{-x}{2\pi \cdot \sigma^{3}\cdot \mu }\cdot e^{-\frac{1}{2}\left(\frac{x^{2}}{\sigma^{2}}+\frac{y^{2}}{\mu^{2}}\right)}.$$

**Figure 4:**
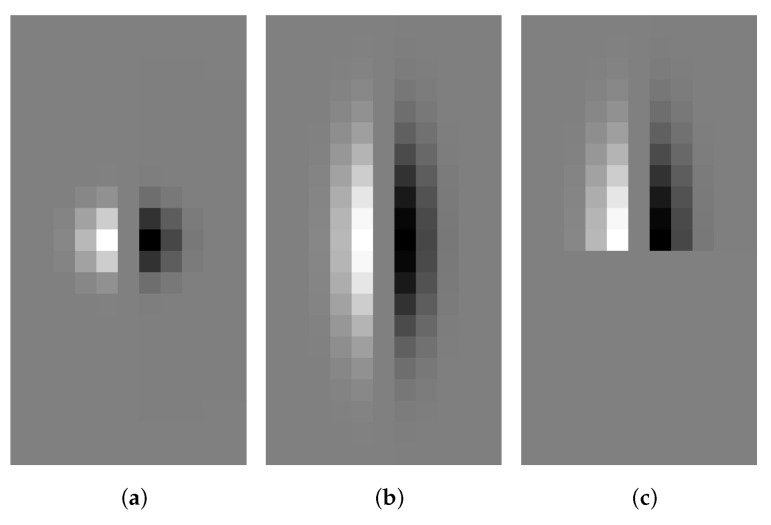
Different discretized 2D derivative Gaussian kernels. (**a**) $G_{\sigma }^{\prime }$, σ = 1, see Equation (3). (**b**) *FOAGK*, σ=1 and μ=3, see Equation (9). (**c**) *HGK*, σ=1 and μ=3, see Equation (10).

An example of the FOAGK kernel is depicted in Figure 4b, alongside the $G_{\sigma }^{\prime }$ shown in Figure 4a. While the $FOAGK_{\sigma ,\mu }$ can be oriented [41], it suffers from a common drawback: it efficiently extracts only one 180${}^{\circ }$-periodic orientation, as detailed and illustrated in [28]. Consequently, these filters face challenges in accurately estimating multiple coexisting orientations at the same pixel.

Contour detection methods, as reported in [29], often encounter reduced accuracy at corner levels and regions of the image containing non-straight structures. To overcome this limitation, a thorough anisotropy analysis in [29] reveal that wedge filters [42,43] or asymmetric oriented filters [30,44] appear to be more suitable, particularly for detecting multiple edge directions or modeling a template. These alternatives provide better capabilities for accurately estimating various coexisting orientations, thereby mitigating the undesirable effects encountered in previous approaches.

Building upon the aforementioned anisotropic filtering assumptions, the proposed technique can effectively extract contours and intersecting corners using two elongated bidirectional filters. The core concept involves symmetrically truncating the anisotropic Gaussian kernel with a Heaviside function and producing various oriented versions (ranging from 0 to 360${}^{\circ }$) of this filter.

The ensued anisotropic detector, based on the HGK derivative, is mathematically defined as follows:

(10) $${HGK}_{\sigma ,\mu }\left(x,y\right)=-H\left(y\right)\cdot FOAGK_{\sigma ,\mu }\left(x,y\right),$$

The function H conforms to the required Heaviside function, as defined in Equation (4). To provide clarity, the ${HGK}_{\sigma ,\mu }$ can be created by combining two 1D components: a semi-Gaussian (truncated Gaussian) and a first derivative of a 1D Gaussian.

These components are defined as follows, for discrete signal *s*:

- a smoothening semi/truncated Gaussian: $G\left(s\right)=H\left(s\right)\cdot e^{\frac{s^{2}}{2\cdot \mu^{2}}}$, with $\mu \in R_{+}^{*}$, s∈R and H is described in Equation (4),
- a first derivative of a Gaussian (derivative of G): $G^{\prime }\left(s\right)=s\cdot e^{\frac{s^{2}}{2\cdot \sigma^{2}}}$, with $\sigma \in R_{+}^{*}$ and s∈R.

To summarize, the ${HGK}_{\sigma ,\mu }$ is formed by combining these two 1D components: the semi-Gaussian G in one direction and the first derivative of a Gaussian $G^{\prime }$ in the other direction.

Figure 4c provides an illustrative example of the ${HGK}_{\sigma ,\mu }$ filter, constructed by applying the $G^{\prime }$ function in the horizontal direction and the G function in the vertical direction. To create an elongated filter, with inherent anisotropy, and achieve significant smoothening in the edge direction for robust edge detection [29,30], it is essential that the support of the smoothing half-filter (G) exceeds that of the filter containing the derivative ($G^{\prime }$), which implies that μ should be greater than σ. Subsequently, to obtain $HGK_{\theta }$ – a rotated version of the filter – the ${HGK}_{\sigma ,\mu }$ filter is directed in multiple directions θ from 0 to $360^{\circ }$. By convolving the image *I* with $HGK_{\theta }$ (i.e., $I*HGK_{\theta }$), derivative information can be computed for each desired direction. It is important to note that when σ=μ, ${HGK}_{\sigma ,\mu }$ is equivalent to $HG_{\sigma }^{\prime }$, resulting in a 2D half isotropic Gaussian filter (described in Equation (4)).

**Figure 5:**
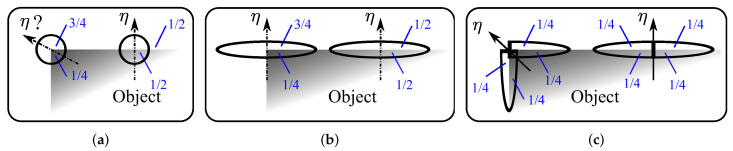
Filter supports’ representation for edges and corners; the $HGK_{\theta }$ enables estimating the two directions of the edges, including corner points. (**a**) Isotropic support ($G_{\sigma }^{\prime }$). (**b**) Anisotropic support ($FOAGK_{\sigma ,\mu }$). (**c**) Half Gaussian Kernels support ($HGK_{\theta }$).

To gain a better understanding of this feature extraction technique [29], let’s consider the supports of both the isotropic ($G_{\sigma }^{\prime }$) and anisotropic ($FOAGK_{\sigma ,\mu }$) filters at a straight contour. In this scenario, both filters are equivalent to 1/2 on both sides of the edge, as depicted in Figure 5a,b. However, at a right-angled corner point ($90^{\circ }$), the supports of the complete filters result in values around 1/4 and 3/4, respectively, on the two sides of the edge, as shown in Figure 5a,b. On the other hand, the support of the oriented half-filter $HGK_{\theta }$ remains constant at 1/2 on both sides of the edge, as illustrated in Figure 5c. This property makes $HGK_{\theta }$ particularly well-suited for accurately estimating edges and corners, as it maintains a consistent response along both sides of the edge, regardless of the orientation. This characteristic is beneficial for robustly capturing edge information in multiple directions, thus enhancing the feature extraction capabilities of the technique.

The $HGK_{\theta }$ filter is an oriented filter, leading to responses that can be either positive or negative, similar to the $HG_{\sigma }^{\prime }$ filter. In line with Equation (6), the gradient |∇I| at each pixel coordinate (x,y) is determined as the difference between the maximum and minimum values of $I*HGK_{\theta }$ across all directions θ:

(11) $$\left\{\begin{array}{ccc} \left|\nabla I|(x,y\right) & = & \max_{\theta \in [0,360^{\circ }[}I*HGK_{\theta }\left(x,y\right)-\min_{\theta \in [0,360^{\circ }[}I*HGK_{\theta }\left(x,y\right),\\ \theta_{1}\left(x,y\right) & = & \underset{\theta \in [0,360^{\circ }[}{\mathrm{argmax}}\left(I*HGK_{\theta }\left(x,y\right)\right),\\ \theta_{2}\left(x,y\right) & = & \underset{\theta \in [0,360^{\circ }[}{\mathrm{argmin}}\left(I*HGK_{\theta }\left(x,y\right)\right),\\ \eta (x,y) & = & \frac{\theta_{1}\left(x,y\right)+\theta_{2}\left(x,y\right)}{2}. \end{array}\right.\;$$

Indeed, for each pixel, the angles $\theta_{1}$ and $\theta_{2}$ are calculated, representing the directions of the contours. These angles are determined based on the maximum and minimum values of $I*HGK_{\theta }$ across all directions θ for that specific pixel. This process is illustrated in Figure 5c. The angles $\theta_{1}$ and $\theta_{2}$ provide valuable information about the orientations of the edges at each pixel location, helping to accurately detect and characterize the contours present in the image.

In summary, our corner analysis involves identifying the directions of maxima in the responses obtained from the $360^{\circ }$ periodically truncated filters. The corner detection process follows the Mehrotra and Nichani method, as detailed in Section 2.2. The key distinction lies in the shape of the filters used. Instead of employing oriented truncated *isotropic* kernels $HG_{\sigma }^{\prime }$, our proposed technique utilizes oriented truncated *anisotropic* kernels $HGK_{\theta }$. The $HG_{\sigma }^{\prime }$ kernels demonstrate high efficiency when dealing with open corner angles (where $\beta \geq 90^{\circ }$), as illustrated in Figure 2. However, the evaluation and results presented in the following section will highlight that the anisotropy of the truncated Gaussian enables reliable corner detection in various types of images. This enhancement in performance allows for more accurate detection of corners with varying angles and shapes.

## 4. Evaluation and Results

In this section, we present a comprehensive evaluation of the corner detection algorithm to assess its performance. Our approach incorporates both qualitative and quantitative measures to provide a thorough analysis of the algorithm’s capabilities as a function of the noise level (Gaussian noise). Moving beyond visual analysis, the widely used root mean square error (RMSE) measure is adopted to further assess the algorithm’s performance. By reporting the RMSE values, a numerical evaluation of the algorithm’s accuracy is provided, taking into account both false positives and false negatives but also the evaluation of pixel distances between the detected corners and ground truth corners. The RMSE approach offers a more precise measure of accuracy by considering the spatial distribution of detection errors. By evaluating the pixel distances from both perspectives—detection to ground truth and ground truth to detection—we obtain a comprehensive understanding of the corner detection algorithms’ performance. Furthermore, we expand our evaluation to include other visual results, showcasing the algorithms’ performance on both synthesized and real images. In parallel, we explore the impact of noise on the results by analyzing RMSE as a function of signal-to-noise ratio (SNR), providing valuable insights into the robustness of the algorithm.

In conclusion, the combination of qualitative and quantitative evaluations provides a holistic assessment of the corner detection algorithms. The inclusion of visual examples and RMSE evaluation offer a thorough exploration of its strengths, limitations, and overall effectiveness.

The Root-Mean-Square Error (RMSE) is computed between the true corners and the extracted features contained in two different binary images. Considering $T_{c}$ and $D_{c}$ the set of true and detected corners, respectively, the equation of the RMSE is given by:

(12) $$\mathrm{RMSE}=\sqrt{\frac{1}{\mathrm{card}\left(T_{c}\right)\;+\;\mathrm{card}\left(D_{c}\right)}\;\cdot \;\left(\sum_{p\in D_{c}}d_{T_{c}}^{2}\left(p\right)\;+\;\;\;\sum_{p\in T_{c}}d_{D_{c}}^{2}\left(p\right)\right)}.$$

For a detected corner *p*∈$D_{c}$, $d_{T_{c}}$(p) represents the minimal Euclidean distance between the pixel *p* and $T_{c}$, whereas if *p*∈$T_{c}$, $d_{D_{c}}\left(p\right)$ corresponds to the minimal distance between *p* and $D_{c}$. Note that the two distances $d_{T_{c}}$ and $d_{D_{c}}$ are recorded for the assessment computation, as detailed in [17]. Indeed, only the calculation of the distances $d_{T_{c}}$ can favor an algorithm where the detected corners are agglutinated around a single true point. Finally, in the proposed experiments, the number of detected corners ($\mathrm{card}(D_{c})$) is the same as the number of the true corners in the ground truth ($\mathrm{card}(T_{c})$); consequently, the compared corner detection methods extract the same number of corners in each image. Note that the RMSE is also also called “localization error”, see [24]. It does not penalize the corners detected very close to their reference, unlike precision/recall-type metrics, which do not tolerate small pixel deviations. Additionally, RMSE is a standard metric that is not only easy to interpret but also very convenient, mathematically. In addition, it is robust to outliers and its symmetry par rapport the over- and under-estimation implies it does not favor one type of error over the other. Finally, to be specific to our problem, the number of the detected corners corresponds to the same number of the ground truth corners, so that the evaluation enables computing errors of both false positive and false negative detections.

**Figure 6:**
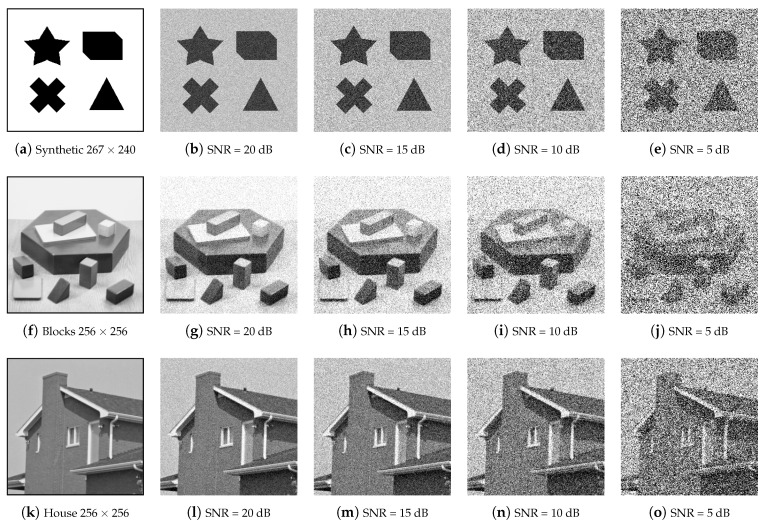
Images utilized in our experimental protocol. The images in (**a**,**f**,**k**) are corrupted by a Gaussian noise where the level of noise is indicated by the SNR value (in decibels—dB—).

Figure 6 represents some of our candidate images as a 3×5 grid with each row containing five different versions of the same image corrupted by Gaussian noise at different noise levels, i.e., Signal-to-Noise Ratios (SNRs) corresponding to 0, 20, 15, 10, and 5 dBs, respectively. The three original images are being identified as Synthetic, Blocks, and House images. In the subsequent figures, for the sake of comparison, we will subject each of these three images, at various SNRs, to five different corner detection approaches, including ours. In addition, we have used the ground truths with corners identified by human inspection. The noisy images are shown as examples only, as we have used various SNRs in our experiments. With ground truth images corresponding to corner identification by inspection, the benchmark methods are described as follows:

1. Kitchen and Rosenfeld defined a cornerness measure for each pixel intensity based on the change of 2nd order gradient direction along the edge weighted by the local gradient magnitude [31]. Note that the convolution with a Gaussian $G_{\sigma }$ (see Equation (2)) is not proceeding with this method but the original image can be smoothed using $G_{\sigma }$ before calculating the cornerness measure.
2. Shi and Tomasi [33] computed the minimum eigenvalue between $\lambda_{1}$ and $\lambda_{2}$ of the symmetric structure tensor M. In this context, they estimated that the corners are primitives, which remain more stable for tracking along a video.
3. Harris and Stephen operator [32], or Plessey operator, is based on principal curvature of local auto-correlation using first-order derivative. This cornerness measure yields two positive values at the corners by computing $Det\left(M\right)-k\cdot {\left(Trace(M)\right)}^{2}$, with k>0.
4. Mehrotra and Nichani [19] is an oriented truncated isotropic Gaussian-based method as detailed in Section 2.

Usually, the area for NMS regarding all the techniques is a square of 7 × 7 size.

**Figure 7:**
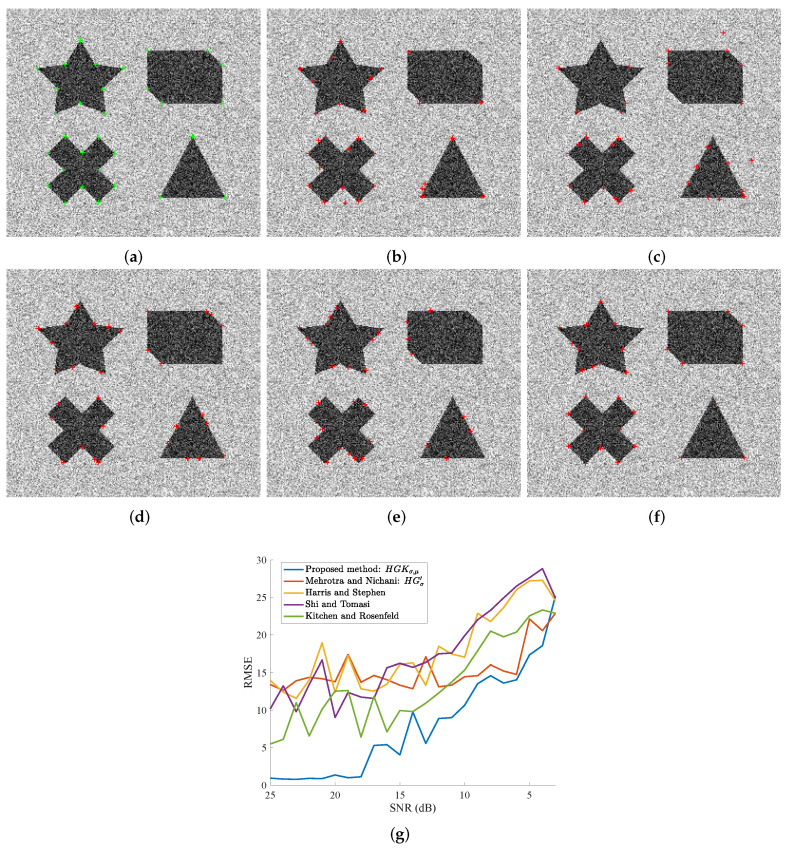
Visual results and scores of the compared methods as a function of the Gaussian noise level (SNR in dB). The displayed image in (**a**) corresponds to a corrupted version of the image in Figure 6a. (**a**) True corners are plotted by green ‘+’ on a noisy image, SNR = 13 dB. In (**c**–**f**), extracted corners are represented by red ‘+’. (**b**) Kitchen and Rosenfeld [31]. (**c**) Shi and Tomasi [33], σ=1 for the tensor. (**d**) Harris and Stephen [32], σ=1 for the tensor. (**e**) Mehrotra and Nichani [19], σ=1 for $HG_{\sigma }^{\prime }$. (**f**) Proposed method, σ = 1 and μ = 3 for $HGK_{\sigma ,\mu }$. (**g**) RMSE scores as a function of the noise level.

Let us first take the synthetic image and observe the performance of our method par rapport with the ground truth and compare it with the likewise results of the benchmarks. Figure 7g plots the RMSE metric (see Equation (12)) against the SNR in dBs, for all the five methods, including our improvements to Mehrotra and Nachani’s method. First, the 31 “best” corners are extracted from this synthesized image by the corner detectors. These corners are composed of acute and obtuse angles. As can be observed, our proposed approach is consistently performing better than the others, unless the noise is very high (less than 5 dB). Without our improvements, the original Mehrotra and Nachani method is outperformed by almost every method, especially at higher SNRs; somewhere between 15 dB and 10 dB it overtakes other methods, but still, our method is better. Note that our method is performing very well given the fact that dB is a logarithmic unit. Visually, we are demonstrating the results related to SNR = 13 dB in Figure 7b–f. A close look will reveal misidentifications in the form of false positives and true negatives, especially with respect to acute corner angles (the star and the triangle) with each of the five methods. With our method, however, the errors are fewer and far less serious in magnitude.

**Figure 8:**
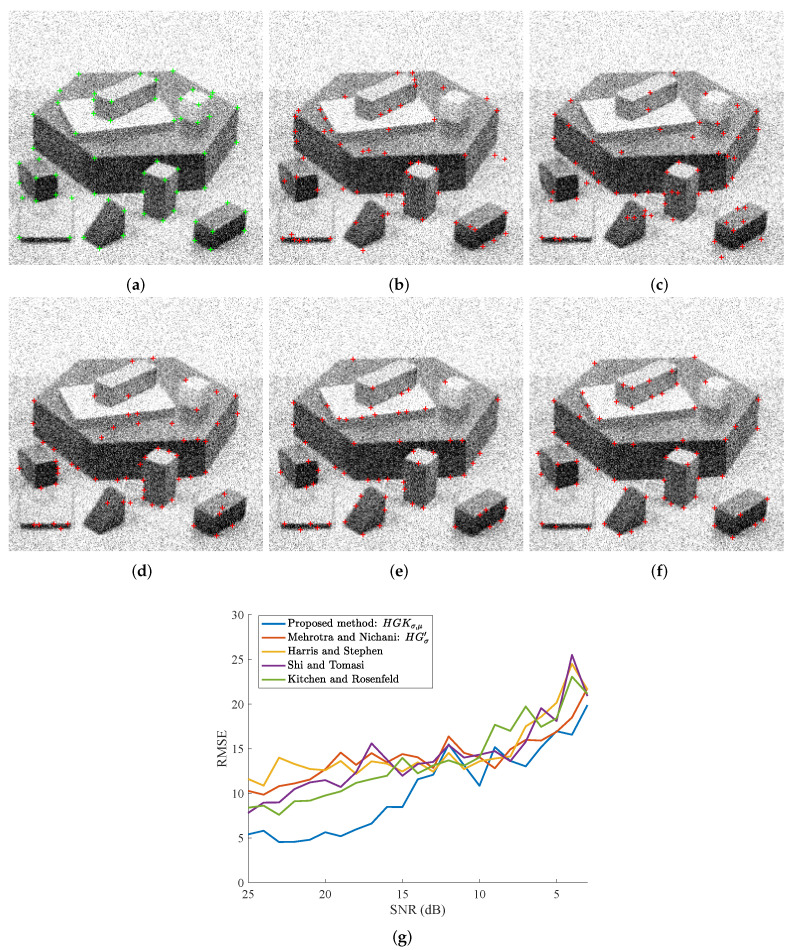
Visual results and scores of the compared methods as a function of the Gaussian noise level (SNR in dB). The displayed image in (**a**) corresponds to a corrupted version of the image in Figure 6f. (**a**) True corners are plotted by green ‘+’ on a noisy image, SNR = 16 dB. In (**c**–**f**), extracted corners are represented by red ‘+’. (**b**) Kitchen and Rosenfeld [31]. (**c**) Shi and Tomasi [33], σ=1 for the tensor. (**d**) Harris and Stephen [32], σ=1 for the tensor. (**e**) Mehrotra and Nichani [19], σ=1 for $HG_{\sigma }^{\prime }$. (**f**) Proposed method, σ = 1 and μ = 3 for $HGK_{\sigma ,\mu }$. (**g**) RMSE scores as a function of the noise level.

The well-known Blocks image resulted in the curves of Figure 8g when subjected to corner detection using the five methods at various levels of Gaussian noise. This image contains 62 referenced corners, which are hand-labelled. In addition, our approach is consistently outperforming others by at least around 12 dB. At very low SNRs too, the proposed method is at par with the better-performing method; sometimes even better. Without our improvements, the original Mehrotra and Nichani method is not performing well on its own. For visual results, we are relying on a ground truth image at 16 dB SNR and as can be observed in Figure 8a–f. Our method identifies far fewer corners as non-corners and vice versa; additionally, the very few misses are by very low margins. Furthermore, the two open corners on the top of the big blocks are both extracted by the proposed method.

**Figure 9:**
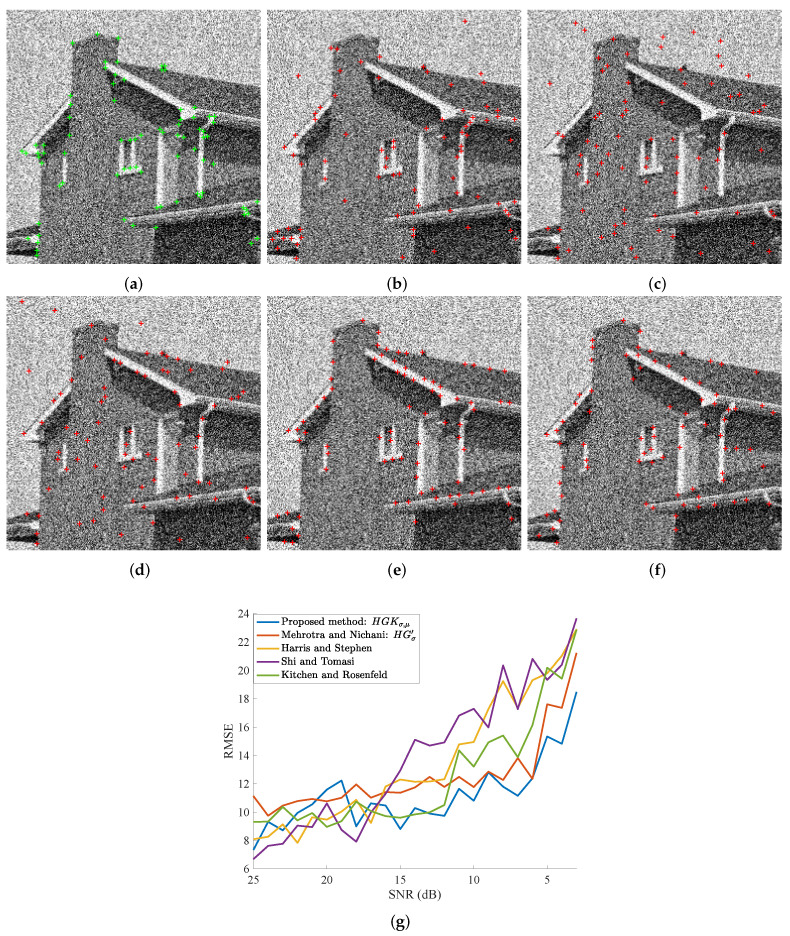
Visual results and scores of the compared methods as a function of the Gaussian noise level (SNR in dB). The displayed image in (**a**) corresponds to a corrupted version of the image in Figure 6k. (**a**) True corners are plotted by green ‘+’ on a noisy image, SNR = 10 dB. In (**c**–**f**), extracted corners are represented by red ‘+’. (**b**) Kitchen and Rosenfeld [31]. (**c**) Shi and Tomasi [33], σ=1 for the tensor. (**d**) Harris and Stephen [32], σ=1 for the tensor. (**e**) Mehrotra and Nichani [19], σ=1 for $HG_{\sigma }^{\prime }$. (**f**) Proposed method, σ = 1 and μ = 3 for $HGK_{\sigma ,\mu }$. (**g**) RMSE scores as a function of the noise level.

With the 256×256 House image, our method is performing better at low SNRs, especially beyond 15 dB, as can be readily observed in Figure 9g. It is competing at higher SNRs but not that well around 18–20 dB. Note that even by manual annotation, the image was hard to handle and likely prone to errors due to the low contrast of the image. This, being our reference (80 hand-labeled corners), may well have caused errors in the corner ascertainment; hence may have led to deviations in the relative results. Overall, however, our method has shown good results, as can be seen in the visual results at 10 dB SNR (Figure 9a–f). Even the best-performing method at high SNRs, in Figure 9g (Shi and Tomasi [33]) too many wayward detections of the corners in the sky part of the image. The same is true of uniform regions like the facade of the building. One cannot notice such waywardness with our method as well as Mehrotra and Nichani’s. Relaxing the RMSE’s assessment may improve the results a lot in our favor.

To provide a more comprehensive understanding of the displayed results, it is important to emphasize the practical implications of the improved corner detection method’s superior performance. This improvement is particularly significant given that dB is a logarithmic unit. Additionally, the new method’s robustness in corner detection under various conditions, as shown by fewer and less severe errors, enhances its applicability in real-world computer vision tasks. The proposed method’s consistent outperformance of benchmark methods, even in challenging conditions, highlights its reliability in corner detection. The visual results at 16 dB SNR underscore its accuracy, reducing the number of non-corners identified as corners and vice versa. The successful extraction of open corners further demonstrates its robustness and precision. Despite the House image’s low-contrast nature, our improved corner detection method excels, particularly at low SNRs. While acknowledging the challenges posed by this image, our results underscore the method’s effectiveness. The visual results at 10 dB SNR reveal our method’s capabilities, even in comparison to the best-performing benchmark, while hinting at future refinements.

In summary, the results of the improved corner detection method exhibit its consistent superiority over classical benchmarks of the era of the original method, especially under challenging conditions. Through the employment of oriented semi/truncated Gaussian-shaped kernels, the method shines both with respect to robustness and accuracy. This is especially pertinent in practical situations where corner detection holds a crucial role across diverse computer vision applications. Furthermore, we acknowledge the unique challenges posed by certain images and offer insights into future avenues for refinement and enhancement, solidifying our commitment to advances in the field of corner detection.

## 5. Conclusions and Contribution

This paper introduced a significant improvement to Merhotra and Nachani’s corner detection method. Our enhancement centers on the concept of half-edges, where corner points are defined as intersections between two half-edges with distinct orientations. Unlike traditional approaches relying on the first derivative of a 2D Gaussian filter, our method leverages anisotropic Gaussian filters. This feature enables the detection of half-edges even in noisy or corrupted images, facilitating the extraction of corners with acute angles. Our experiments, conducted on synthetic and real images, consistently demonstrated the efficiency and reliability of the proposed algorithm. Notably, our results surpassed those of well-established corner detection benchmarks, showcasing the transformative impact of our modifications. The improved corner detection process, characterized by its ease of implementation, has the potential to become a valuable reference for the computer vision community tackling corner detection challenges.

The primary contribution of this study lies in the significant improvement we have introduced to the field of corner detection. Our method, based on the concept of half-edges and anisotropic Gaussian filters, addresses key limitations in traditional corner detection algorithms. By enabling the detection of corners with acute angles and enhancing robustness in noisy or corrupted images, our approach offers a substantial advancement. The significance of our study extends to several dimensions within the corner detection research community. First, it advances the state-of-the-art in corner detection, demonstrating superior performance in comparison to well-established benchmark methods. This enhanced accuracy and reliability open doors to applications in diverse domains, including robotics, autonomous navigation, structural analysis, and medical imaging. Second, our approach presents a valuable addition to the toolkit of computer vision practitioners. Its ease of implementation and superior results make it an attractive option for researchers and engineers seeking robust corner detection solutions. Lastly, our study contributes to the ongoing dialogue in computer vision by showcasing the potential of anisotropic Gaussian filters and half-edge concepts. We hope to inspire further research into the optimization and adaptation of these principles for various computer vision challenges.

Looking ahead, future research in this domain could explore several promising avenues. First, further optimization and fine-tuning of our method may lead to even more robust and accurate corner detection, particularly in complex real-world scenarios. Additionally, investigating applications in fields such as robotics, autonomous navigation, and object recognition could uncover new use cases and refine the algorithm’s practicality. Furthermore, addressing the computational efficiency and scalability of our approach for large-scale image datasets is an important consideration. As computer vision continues to evolve, the demand for efficient corner detection algorithms that can handle big data becomes increasingly pressing.

In a nutshell, our work not only advances corner detection but also opens doors to broader applications in computer vision. By continually refining and adapting this method, we can anticipate its continued relevance and impact in the ever-expanding field of computer vision.

## Figures and Tables

**Table 1:** $HG_{\sigma }^{\prime }$ shape as a function of the σ parameter and the spacial support.

$HG_{\sigma }^{\prime }$ spacial support (in *x*)	3	5	7	9	11	13	15	17	19
σ value	0.7	1.11	1.53	1.95	2.38	2.8	3.23	3.66	4.09
$HG_{\sigma }^{\prime }$ displayed in 2D (images 25 × 25)	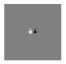	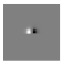	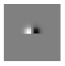	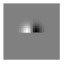	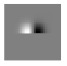	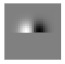	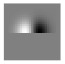	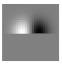	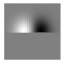

## Data Availability

The data and the Matlab code can be made available by writing an email to Dr. Baptiste Magnier.
